# At the tipping point: Differential influences of warming and deoxygenation on the survival, emergence, and respiration of cosmopolitan clams

**DOI:** 10.1002/ece3.4041

**Published:** 2018-04-19

**Authors:** Tae Won Kim, Shinyeong Park, Eunchong Sin

**Affiliations:** ^1^ Department of Ocean Sciences Inha University Incheon Korea; ^2^ School of Biological Sciences Seoul National University Seoul Korea; ^3^ Division of Polar Ocean Sciences Korea Polar Research Institute Yeonsu‐gu, Incheon Korea; ^4^ Department of Polar Sciences University of Science and Technology Yuseong‐gu, Daejeon Korea

**Keywords:** dissolved oxygen, emergence, Manila clams, mortality, respiration, warming

## Abstract

Although warming and low dissolved oxygen (DO) levels are co‐occurring significant climatic stressors in the ocean, the combined effects of these stressors on marine benthic animals have not been well established. Here, we tested the effects of elevated temperatures and low dissolved oxygen levels on the survival, emerging behavior from sediment, and the respiration of juvenile cosmopolitan Manila clams (*Venerupis philippinarum*) by exposing them to two temperatures (20 and 23.5°C) and DO levels (3.5 and 6–7 mg/L). Although within previously described tolerable ranges of temperature and DO, this 3.5°C increase in temperature combined with a 50% decrease in DO had a devastating effect on the survival of clams (85% mortality after 8 days). The mortality of clams under normoxia at 23.5°C appeared to be higher than under the low DO condition at 20°C. On the other hand, more clams emerged from sediment under the low DO condition at 20°C than under any other conditions. Oxygen consumption rates were not significantly affected by different conditions. Our results suggest temperature elevation combined with low oxygen additively increases stress on Manila clams and that warming is at least as stressful as low DO in terms of mortality. However, low DO poses another threat as it may induce emergence from sediment, and, thus increase predation risk. This is the first evidence that a combination of warming and deoxygenation stressors should reduce population survival of clams much more so than changes in a single stressor.

## 1 INTRODUCTION

Global temperatures are predicted to increase by 3–4°C by the end of this century according to the Intergovernmental Panel on Climate Change scenario (IPCC, 2014). As a result, ocean temperatures are expected to increase accordingly (Belkin, 2009; Keeling, Kortzinger, & Gruber, 2010). Ocean warming is not the only stressor of marine organisms because oxygen solubility will decrease as temperatures increase (Keeling & Garcia, 2002; Keeling et al., 2010). The resulting deoxygenation will cause problems for marine animals. Higher temperatures will also increase the metabolisms of marine organisms and facilitate phytoplankton blooms that will increase hypoxia during subsequent decomposition (Brewer & Peltzer, 2016; Praetorius et al., 2015). Therefore, temperature increases in water cause oxygen depletion, and in combination, these two stressors influence the behaviors and physiologies of marine animals (Portner, 2012; Rosa & Seibel, 2008).

A number of studies have been conducted on the effect of increases in water temperature on marine animals (e.g., Kim & Micheli, 2013; Moline, Claustre, Frazer, Schofield, & Vernet, 2004; Portner, 2002). Notably, the physiological processes of ectothermic animals are dependent on temperatures remaining within optimal ranges (Newell, 1966; Portner, 2012). When temperatures increase beyond optimal ranges, animals fail to adapt or acclimate and may eventually become extinct (Portner, 2002; Rummer et al., 2014). Likewise, the effects of low dissolved oxygen (DO) levels on marine animals have been well investigated (Conley, Carstensen, Vaquer‐Sunyer, & Duarte, 2009; Diaz & Rosenberg, 1995; Kim, Barry, & Micheli, 2013; Wu, 2002). Generally, many benthic animals do not cope well with hypoxia (<2 mg/L DO) and eventually die (Vaquer‐Sunyer & Duarte, 2008). As compared with the number of studies conducted on the influences of temperature increases or hypoxia, the combined effects of increases in temperature and low DO levels in a controlled environment have been far less studied despite their relevance to marine ecosystems (Cheng et al., 2015; Vaquer‐Sunyer & Duarte, 2011).

Here, we explored the effects of an increase in temperature and a reduction in DO level on a major fishery and aquaculture resource, the Manila clam *Venerupis philippinarum*. This species originated in Asia and was then maricultured in America and Europe and is now one of the most popular marine food sources worldwide (Becker, Barringer, & Marelli, 2008; Cordero, Delgado, Liu, Ruesink, & Saavedra, 2017). *V. philippinarum* lives in intertidal or subtidal areas that experience considerable fluctuations in abiotic factors, including temperature and oxygen. We exposed juvenile Manila clams to combinations of two temperatures (20 and 23.5°C) and two DO levels (3.5 and 6–7 mg/L), which model conditions frequently experienced (Kozuki et al., 2013; Shin, Kim, Chung, & Hur, 2000; Uzaki, Kai, Aoyama, & Suzuki, 2003), to determine the effects of combined temperature and DO changes on mortality, emerging behavior from sediment, and oxygen consumption rates. Because clams generally burrow into sediment to escape predation, emerging behavior is a behavioral response to stressors, such as hypoxia and hypercapnia (Lee & Kim, 2017; Lee, Lee, & Chin, 2012; Saloom & Duncan, 2005).

We predicted clam survival would not be seriously influenced by temperature and DO changes within their tolerable ranges, but that emerging response from sediment and oxygen consumption rates would be influenced by both stressors. In addition, we predicted oxygen consumption rate, an indicator of metabolic activity, would be influenced by temperature and DO changes.

## 2 MATERIALS AND METHODS

### 2.1 Mortality and emergence

For the experiment, approximately 600 Manila clams were collected from an intertidal mudflat in Jugyo‐ri, Jugyo‐myeon, Boryeong‐si, Chungcheongnam‐do, Republic of Korea, on 3 August 2015. They were immediately placed in an ice box without seawater and transferred to a well‐aerated acclimation aquarium (500 L, 150 cm × 70 cm × 80 cm, W × L × H) in a laboratory at the Korea Institute of Ocean Science and Technology (KIOST) (Ansan, Korea). Water temperature and salinity were maintained at 20°C and at 33 psu, respectively. The mean annual temperature of seawater at Jugyo‐ri is approximately 21°C, and the optimal temperature range for Manila clams is known to be 18 to 23°C, as determined by clearance rates (Kang et al., 2016). Experimental temperatures were regulated using a chiller and/or heater connected to a thermostat. Salinity was controlled by adding unrefined sea salt or distilled water to seawater in accord with saltmeter (YSI‐30) readings. Clams were fed sufficiently once every 2 days at 50 ml of 1:10 diluted Sellfish Diet 1800^®^ (Reed Mariculture Inc., USA, two billion cells per 10 ml) per 80 individuals. The photoperiod used matched the natural light: dark (L:D) cycle outside the laboratory. The clams were acclimated to laboratory condition for 7 days.

For the experiment, we selected 400 healthy juvenile manila clams of similar size (15.99 ± 1.28 mm shell length). Ten clams were allocated randomly to each of forty 500‐ml transparent glass jars containing 3 cm of sediment collected at the same Jugyo‐ri intertidal mudflat where the clams were collected. Clams in each jar were marked from 1 to 10 on both sides of the shell with a Monami^®^ permanent marker pen for individual identification. Ten randomly selected jars were assigned to each of four acrylic tanks (80 cm × 75 cm × 20 cm) maintained under the same conditions for acclimation. The water in each tank was maintained at 20°C and 33 psu and was passed through two filters and a cooler (DAEIL, DBA‐075 or JEIO TECH, RW‐3025G Jeio Tech^®^) before being delivered independently to jars at 75 ml/min using a 5‐mm‐diameter hose. Water in each jar overflowed separately into a holding tank. Room temperature was maintained at ~21.5°C using an air conditioner. The photoperiod followed a 12/12‐hr light/dark cycle.

The experiment was conducted using a 2 × 2 matrix design at temperatures of 23.5°C (high) or 20°C (normal) and DO levels of 6–7 mg/L (85%–100%; normoxia) or 3.5 mg/L (40%–50%; low oxygen). Clams were gradually exposed to the higher temperature by increasing water temperature from 20 to 23.5°C at a rate of 0.5°C daily using a RW‐3025 Jeio Tech^®^ heater. The low target DO level (3.5 mg/L) was achieved at water temperatures of 20 and 23.5°C by mixing N_2_ gas with air at an approximate volume ratio of 4:1 (N_2_ gas = 400 cc/s, air = 100 cc/s) using flow meters. An ambient salinity of 33 psu was maintained throughout during all four experimental conditions.

Clams were maintained under the four experimental conditions for 8 days. Because over 80% succumbed to the high temperature/low DO treatment after 8 days, experimental exposure was terminated at this time. Temperature and DO were measured at 9:00 a.m. and 6:00 p.m. daily using a YSI 30 or a YSI 5000, respectively. The higher temperature was maintained at 23.60 ± 0.21°C under normoxia and at 23.46 ± 0.18°C under low DO conditions, and low temperature was maintained at 20.31 ± 0.19°C under normoxia and 20.25 ± 0.28°C under low DO conditions. Normoxia was maintained at 7.32 ± 0.87 mg/L at high temperature and at 7.90 ± 0.96 mg/L at low temperature, and low DO was maintained at 3.65 ± 0.31 mg/L at high temperature and at 3.91 ± 0.63 mg/L at low temperature.

Animal status (live/dead) was checked in each jar at 10:00 a.m. daily during the experimental period. Individuals that did not close in response to touch or were too light were considered dead, and removed from jars. Mortality (%) was calculated by expressing the number of dead individuals as a percentage of initial individuals. The locations of each clam in each jar (in/out of sediment) were also checked daily. Emergence from sediment was interpreted as a significant response to stressors (Lee & Kim, 2017), and emergence rates (%) were calculated by expressing the number of clams that emerged from sediment as a percentage of live individuals.

### 2.2 Respiration

In this experiment, we used approximately 300 Manila clams collected from an intertidal mudflat at Namdang‐ri, Hongseong‐gun, Chungcheongnam‐do, Republic of Korea, in 19 August 2016. They were immediately placed into an ice box and transferred to 2 well‐aerated acclimation water baths (575 mm × 415 mm × 255 mm, W × L × H) in an incubator at KIOST. The incubator temperature was set at 20°C, and a salinity of 33 psu was maintained by adding unrefined sea salt or distilled water as determined using a salt meter (YSI‐30, YSI Inc., USA). Clams were fed sufficiently once every 2 days with one ml of Reef Zooplankton^TM^ (Seachem, USA). The incubator was not illuminated.

After acclimation for 5 days, 196 healthy juvenile clams (17.45 ± 1.14 mm in shell length, 7.94 ± 0.66 mm in shell height, and 1.08 ± 0.23 g in wet weight including shells) were selected randomly for the experiment. These clams were numbered 1–196 with Monami^®^ permanent marker pen, and seven clams were allocated randomly to each of 28 transparent glass jars (1,200 ml). Seven randomly selected jars were then assigned to each of four aquariums (= seven clams × seven jars × four treatments). Experimental treatment water was supplied as mentioned above in the previous experiment. Each jar was supplied with a stream of water (200 ml/min). All experimental aquariums were aerated continuously, and water environments were kept clean.

In the high temperature group, the temperature was gradually increased in situ to 23.5°C at rate of 2°C over the first 24 hr and of 1.5°C over the next 24 hr. A heater (RW‐3025 Jeio Tech^®^) was used to increase water temperature and chillers (DAEIL^®^, DBA‐075) were used to reduce water temperature. After the targeted water temperature had been reached, the low oxygen level of 3.5 mg/L was obtained by mixing N_2_ (800 cc/min) using gas flow meters (MFC Korea^®^) and Witrox control software (Loligo^®^ system, USA). Ambient salinity was maintained at 33 psu.

Experiments under different temperature and dissolved oxygen level conditions were conducted over 4 days. Dissolved oxygen and temperature were monitored every second using Witrox control software (Loligo^®^ system, USA). During this experiment, high temperature was maintained at 23.40 ± 0.21°C under normoxia and at 23.49 ± 0.11°C under low DO, and low temperature was maintained at 20.05 ± 0.29°C under normoxia and at 19.70 ± 0.73°C under low DO. Normoxia was maintained at 6.72 ± 0.22 mg/L at high temperature and at 7.27 ± 0.25 mg/L at low temperature, and low DO was maintained at 3.57 ± 0.27 mg/L at high temperature and at 3.52 ± 0.30 mg/L at low temperature. Salinity and pH were measured using a salt meter (YSI‐30, YSI Inc., USA) and a pH meter (Mettler Toledo^®^ SevenMulti) each morning during the entire experimental period. Jars were checked daily for dead clams.

Oxygen consumption rates were measured three times: 3 days before, 1 day after, and 4 days after exposure to the different treatments. Three clams were selected randomly from each jar and transported to a chamber (500 ml) connected to an optical dissolved oxygen sensor and filled with water that matched the conditions clams had been subjected to. For respirometry, an autorespirometry system (Loligo^®^ systems) was used and the oxygen consumption rates of three randomly selected clams were measured for 30 min using the closed respirometry method. Oxygen consumption rate was defined as the amount of oxygen consumed per kg of wet weight per hour (mg O_2_ kg^−1 ^hr^−1^). After completing the experiments, all soft tissues were scraped from shells and dried at 80°C for 24 hr in an oven, and oxygen consumption rates were then recalculated on a dry weight basis. Measurements were conducted in the dark and feeding was stopped 2 days before measurements to prevent metabolic activity induced by light or digestion.

### 2.3 Statistical analysis

All proportional data (cumulative mortalities and emergence rates) were normalized by arcsine‐root‐square transformation. Two‐way ANOVA was used to determine whether temperature and/or DO influenced mortality. Repeated‐measures two‐way ANOVA was performed to determine whether temperature, DO, or/and exposure time (days) influenced emergence rates. When the assumption for equal between‐group correlations and group variances (“sphericity”) was violated (Mauchly's test, *p* < .05), Huynh–Feldt corrections were applied. Oxygen consumption rate data were only used when the coefficient of determination *r* ^2^ was >.25. Because oxygen consumption rate might have been dependent on time of measurement, endogenous respiration rhythm (circadian and/or circatidal rhythms) they were compared by two‐way ANOVA at each measurement time. The statistical analysis was conducted using SPSS ver. 24.0.

## 3 RESULTS

### 3.1 Mortality and emergence

**Figure 1:**
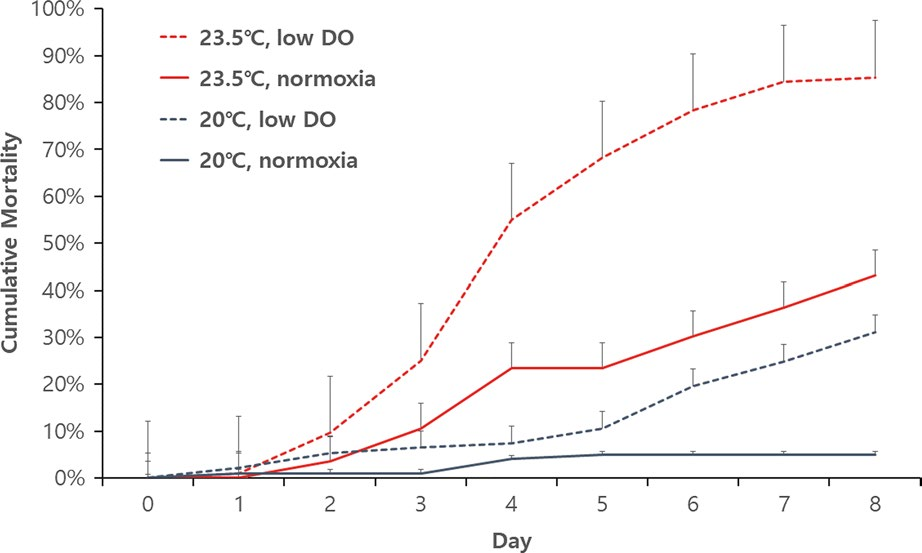
Cumulative mortality of clams at different temperatures and DO levels (mean ± *SE*)

Both temperature increase and oxygen depletion significantly increased clam mortality (Two‐way ANOVA, temperature: *F*_1,36_ = 157.892, *p* < .001, DO: *F*_1,36_ = 72.456, *p* < .001, Figure 1). However, the interaction between temperature and DO (*F*_1,36_ = 2.209, *p* = .146) did not have a significant influence on mortality.

**Figure 2:**
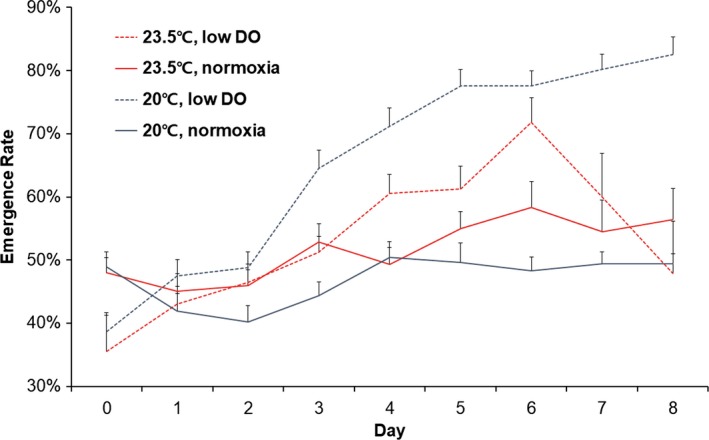
Percentages of clams emerging from sediment at different temperatures and DO levels (mean ± *SE*)

Although emergence rate appeared to increase under low DO conditions, DO (*F*_1,34_ = 3.732, *p* = .062) and temperature (*F*_1,34_ = 1.547, *p* = .222) had no significant effect on emergence rate (Figure 2). However, time (*F*_4.968,168.9111_ = 8.074, *p *<* *.001) and the interaction between time and DO (*F*_4.968,168.9111_ = 2.204, *p* = .05) did significantly influence emergence rate. On the other hand, interactions between time and other factors had no significant effect on emergence rate (Time × Temperature: *F*_4.968,168.9111_ = 1.277, *p* = .276, Time × Temperature × DO: *F*_4.968,168.9111_ = 0.583, *p* = .712).

### 3.2 Respiration

**Figure 3:**
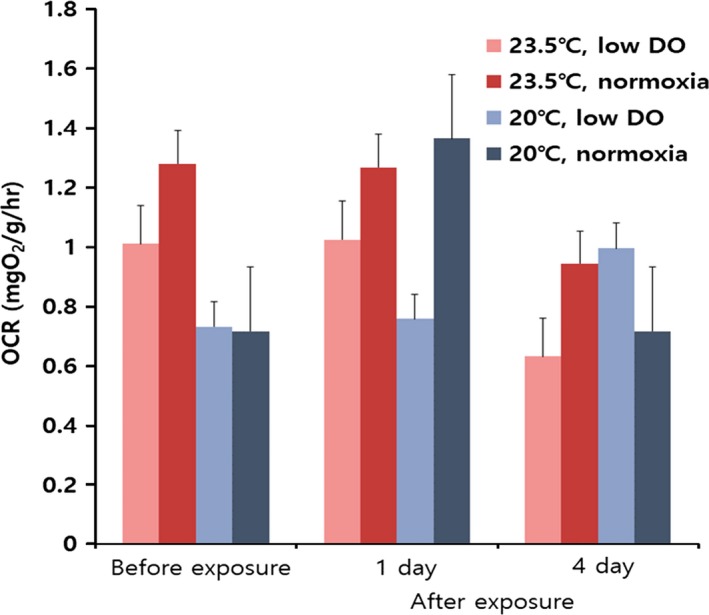
Oxygen consumption rates at different temperatures and DO levels (mean ± *SE*)

Temperature increase and/or decrease in DO had no significant effect on respiration before (*F*_3,18_ = 0.712, *p *=* *.558) or after exposure (1st measurement: *F*_3,18_ = 1.113, *p* = .370, 2nd measurement: *F*_3,16_ = 0.493, *p* = .692) (Figure 3).

## 4 DISCUSSION

Increasing temperature and decreasing DO level within known tolerable ranges (Kang et al., 2016; Shin, Kim, Chung, & Hur, 2001; Shin et al., 2000) had a surprisingly devastating effect on Manila clams. In the present study, clam mortality was significantly higher under the high temperature plus low DO condition than under any other condition, and more than 80% of clams died after 8 days of exposure. Although not significantly different, mortality under the high temperature plus high DO condition appeared to be higher than under the low temperature plus low DO treatment. Furthermore, low DO significantly increased emergence rate in a time‐dependent manner. Unexpectedly, respiration was not found to be significantly affected by the different treatments. Our results suggest that temperature increase and DO decrease act as independent significant stressors in terms of mortality and emerging behavior.

The interaction between temperature and DO did not have a significant effect on the mortality of Manila clams. However, given that mortality was highest under the high temperature/low DO condition, temperature and DO did appear to additively influence clam mortality. Our results indicate that low DO level (3.5 mg/L), which is experienced more frequently than hypoxic conditions (DO 2 mg/L), can also seriously influence clam populations. More interestingly, although 23.5°C is within the temperature range required for Manila clam culture (Kang et al., 2016; Shin et al., 2000), a 3.5°C temperature increase from 20°C appeared to be more stressful than a 50% decrease in DO in terms of mortality. Probably, juvenile clams are more sensitive to temperature increases than adults, which have been shown to be tolerant to temperature increases (Kang et al., 2016; Shin et al., 2000). It was surprising to find that the 3.5°C temperature increase work at least the same level to the low DO condition. In a related study, Manila clams at 15°C were found to be tolerant to hypoxia as survival was not affected at DO levels of <l.0 mg/L (Kim, Buck, & Barry, in preparation). Taken together, temperature increases might be more stressful than DO, because they narrow the tolerable DO range. Furthermore, temperature increases in seawater are naturally accompanied by DO decreases, and thus, temperature increases doubly stress Manila clams.

Low DO levels were also observed to increase clam emergence from sediments, which indicates DO rather than temperature acts as a significant stressor in terms of emergence behavior. The reduced emergence rate observed during the latter part of the experimental period under high temperature/normoxia conditions was probably due to high mortality. Given that Manila clams showed higher emergence rates when CO_2_ levels increased or pH levels decreased in a previous study (Lee & Kim, 2017), it appears emergence from sediment is a response to low oxygen and high CO_2_ levels. To resolve oxygen deficiency issues in sea water, clams may emerge from sediment to breath more efficiently, but emergence from burrows probably increases vulnerability to predators (Howard, Poirrier, & Caputo, 2017; Seitz, Marshall, Hines, & Clark, 2003; Taylor & Eggleston, 2000). In previous studies, higher clam emergence rates were associated with increased clam consumption rates by crabs for Manila clams (Kim & Barry unpublished data) and other clam species (Howard et al., 2017; Seitz et al., 2003; Taylor & Eggleston, 2000).

However, in the present study, respiration activities did not differ between treatments, and thus, clams appear to maintain constant oxygen consumption rates regardless of temperature and DO level. In another study, adult Manila clams showed no changes in oxygen consumption rate at DO levels ranging from 6.5 to 3.5 mg/L (Shin et al., 2001). Generally, clams exhibit elevated oxygen consumption rates at high temperatures and low oxygen consumption rates when low dissolved oxygen levels are low (Kang et al., 2016; Lee et al., 2012). Probably, other physiological process such as temperature‐induced hypoxemia, oxidative damage, and apoptosis in the gills largely determined clam survival (Clark et al., 2013; Portner, 2012; Yin, Chen, Chen, Jin, & Yan, 2017).

It is generally believed that increase in temperature decreases hypoxia thresholds for many species, and therefore, high temperature and hypoxia act as detrimental stressors (Portner, 2010; Vaquer‐Sunyer & Duarte, 2011). However, in a previous study, a temperature increase was found to enhance Olympia oyster growth, whereas hypoxia had a detrimental effect (Cheng et al., 2015). Our results also suggest that although high temperature and low DO in combination have a detrimental effect on Manila clams in terms of mortality and that these two stressors affect clam physiology in different ways. Taken together, our findings suggest concomitant increases in temperature and decreases in DO may differentially influence benthic animal physiologies in a species‐dependent manner.

In conclusion, both ocean warming and low oxygenation may seriously influence the survival of Manila clams, but each of these climatic stressors differentially influences this species. A combination of warming and hypoxic stressors is expected to reduce the likelihood of population survival much more so than changes in a single stressor. Global warming is not an independent stressor as it also causes oxygen deficiencies in local environments, and thus, these stressors should be considered in combination.

## CONFLICT OF INTEREST

None declared.

## AUTHOR CONTRIBUTIONS

TWK designed and conducted the experiment, and wrote the manuscript. SP conducted the experiment, analyzed the result, and wrote the manuscript. ES conducted the experiment, analyzed the result, and wrote the manuscript.
